# CONSTRUCT VALIDITY OF FRABONI SCALE OF AGEISM IN A CHINESE-MAJORITY SAMPLE FROM SINGAPORE

**DOI:** 10.1093/geroni/igad104.0878

**Published:** 2023-12-21

**Authors:** Yuanyuan Cao, Jie Xin Lim, Ringo Ho, Yin-Leng Theng

**Affiliations:** Nanyang Technological University, Singapore, Singapore; Nanyang Technological University, Singapore, Singapore; Nanyang Technological University, Singapore, Singapore; Nanyang Technological University, Singapore, Singapore

## Abstract

The 29-item Fraboni Scale of Ageism (FSA; Fraboni et al., 1990) was constructed to measure three dimensions of ageism (Antilocution, Discrimination, and Avoidance) using samples from Canada. The factor structure of the FSA has been challenged in recent studies using US samples (Rupp et al., 2000) and Chinese samples (Fan et al., 2020), resulting in alternative factor structures. The current study aimed to test the different factor structures proposed in these past studies with a sample from Singapore, a Chinese-majority multicultural country. Data from 311 individuals, aged between 21- and 55-year-old were collected. They completed the 29-item FSA using a six-point agreement scale. Confirmatory factor analysis was used to test the goodness-of-fit of the aforementioned factor structures. The results indicated that none of three models provided a good fit to the data. A follow-up exploratory factor analysis with parallel analysis suggested a 3-factor structure. Thirteen out of the 29 items were found to have at least one salient cross-loading after Geomin rotation. These findings suggest that the samples likely interpreted and responded to the items differently resulting in different item-factor configurations, implying the impact of regional culture on the construct validity of FSA. Researchers aiming to quantify ageism in their population using FSA are advised to examine the FSA factor structure prior to using it.

